# Management of pressure injuries with associated osteomyelitis in people with spinal cord injury: a national survey of referral centers in France

**DOI:** 10.1038/s41393-025-01084-y

**Published:** 2025-05-10

**Authors:** Hélène Le Liepvre, Emma D’Anglejan, Haude Chaussard, Damien Marmouset, Killian Allepot, Frédérique Bouchand, Latifa Noussair, François Genêt, Vincent T. Carpentier, Aurélien Dinh

**Affiliations:** 1 AP-HP. Paris Saclay University, Raymond Poincaré Hospital, Department of Physical Medicine and Rehabilitation, Pressure Injury Treatment Unit (UPOH), U1179 END-ICAP, INSERM, Versailles Saint-Quentin-en-Yvelines University (UVSQ), Paris Saclay University, Garches, France; 2 AP-HP. Paris Saclay University, Raymond Poincaré Hospital, Department of Infectious Disease, IHU PROMETHEUS, Versailles Saint-Quentin-en-Yvelines University (UVSQ), Paris Saclay University, Garches, France; 3 AP-HP. Paris Saclay University, Raymond Poincaré Hospital, Department of Orthopedic Surgery, Department of Plastic and Reconstructive Surgery, Versailles Saint-Quentin-en-Yvelines University (UVSQ), Paris Saclay University, Garches, France; 4 AP-HP. Paris Saclay University, Raymond Poincaré Hospital, Department of Pharmacy, IHU PROMETHEUS, Versailles Saint-Quentin-en-Yvelines University (UVSQ), Paris Saclay University, Garches, France; 5 AP-HP. Paris Saclay University, Raymond Poincaré Hospital, Laboratory of Microbiology, IHU PROMETHEUS, Versailles Saint-Quentin-en-Yvelines University (UVSQ), Paris Saclay University, Garches, France

**Keywords:** Spinal cord diseases, Bacterial infection

## Abstract

Pressure injuries are common among individuals with spinal cord injury and often complicated by superinfection, leading to sepsis, hospitalizations, and significant healthcare costs. Osteomyelitis associated with pressure injuries poses diagnostic challenges and requires effective medical and surgical management. To investigate current practices, a national survey of French centers managing over 20 cases annually was conducted. Results revealed significant variability in surgical techniques, antibiotic protocols, and people care pathways. Musculocutaneous and perforator flaps were commonly used, but their application differed among centers. No flaps were performed during acute infections due to failure risks. Bedridden periods and hip flexion protocols varied, with prolonged immobility linked to complications such as thromboembolism and malnutrition. Antibiotic durations ranged from 5–180 days, reflecting differing approaches to infection management. Cure rates within one year ranged from 70–90%. These findings underscore the need for standardized, evidence-based protocols to optimize care for this vulnerable population.

## Background

Pressure injuries are a significant and lifelong complication among individuals with spinal cord injury (SCI) [1], impacting their physical, psychological, and social well-being as well as overall quality of life [2]. Pressure injuries are common and exhibit a wide clinical spectrum. They are frequently complicated by superinfection, which can progress to sepsis, necessitating multiple hospitalizations and prolonged lengths of stay [2]. Furthermore, these injuries impose a substantial economic burden on healthcare systems. Prevention is therefore crucial, requiring the identification and mitigation of associated risk factors [2].

Osteomyelitis associated with pressure injuries is relatively uncommon, but its diagnosis remains challenging. Life-threatening infections can occur and may require immediate surgical debridement. Conversely, surgical fasciocutaneous coverage procedures are often delayed, depending on the extent and progression of the injury and the feasibility of intervention [3]. In such cases, adequate debridement followed by coverage with a flap containing muscle or fascia is recommended [3]. Despite the clinical importance of osteomyelitis in the context of pressure injuries, comprehensive epidemiological, pathophysiological, and clinical data are lacking. Moreover, there are no clinical trials or comparative studies addressing key aspects of management, such as the optimal duration of antimicrobial treatment, the choice of specific drug classes, or the administration route (oral versus parenteral) [4]. As a result, management strategies heavily rely on expert opinion.

Selecting the appropriate antimicrobial therapy is crucial, but this population presents unique challenges due to their high exposure to antibiotics. This predisposition leads to a high rate of colonization and infections caused by multidrug-resistant organisms (MDROs) [5]. Consequently, careful consideration is required to balance effective treatment with the risk of exacerbating antimicrobial resistance.

Many questions remain unanswered regarding the management of osteomyelitis associated with pressure injuries. This uncertainty contributes to significant variability in clinical practice, as highlighted by the limited data and surveys reported to date [2, 6–9]. To provide further insights into current clinical practices, we conducted a national survey among expert centers in France.

## Methods

To address these questions, we identified the referral French centers managing over 20 people annually who undergo fasciocutaneous coverage procedures. This identification was conducted using the national medical information system program. We conducted an electronic survey at each center to collect data on their organization and management practices between 2020 and 2023. The survey comprised three main sections:

1. General Information:
  This section provided a detailed description of the hospital, including the annual number of visits and staff composition. Data were collected on the types and numbers of healthcare workers and medical doctors involved, the volume of surgeries performed, cure rates, people profiles, types of fasciocutaneous coverage/flaps used, and discharge conditions.
2. Preoperative Assessment and Rehabilitation Medicine:
  This section focused on preoperative evaluations, such as ergotherapy consultations, urological examinations, and nutritional assessments. It also included information on postoperative protocols, covering aspects like seating assessments, specialized equipment, and postoperative care procedures.
3. Infectious Disease Management:
  The third section addressed infection-related practices, including postoperative antibiotic treatment, treatment duration, and the use of suction drainage cultures.

The survey consisted of 40 questions, including 19 open-ended questions (see Supplementary Information). Cure rate was defined as the absence of recurrence within one year.

## Results

The survey received exhaustive responses from six centers managing more than 20 people annually (Table 1). The main centers were Berck Local Hospital and Garches University Hospital, conducting over 150 surgeries per year. Preoperative evaluations across all centers involved a multidisciplinary team, including surgeons, physical medicine and rehabilitation specialists, nutritionists, and extended sitting recovery programs, resulting in a homogeneous patient population. The mean age of people ranged from 42–56 years, with a male-to-female ratio of 3. The predominant neurological history was spinal cord injury, accounting for 72–98% of cases.

**Table 1:** Characteristics of people and the management of their pressure injury related osteomyelitis according to hospital.

	All	Berck public hospital	Garches university hospital	Montpellier university hospital	Nantes university hospital	Toulouse university hospital	Marseille private hospital
Centers characteristics							
Surgeries per year (in number)	40 (30; 80)	190	150	80	40	30	20
People’s characteristics							
Mean age (in years)	50 (6)	56	55	42	48	MD	50
Sex ratio (M/F)	3 (1)	3	3	2	4	MD	4
Spinal cord injury (in %)	85 (12)	72	80	98	78	MD	97
Characteristics of the management of pressure injury related osteomyelitis							
Mean length of stay in surgical ward (in days)	8 (6; 10)	6	10	6	0	9	12
Mean duration of strict postoperative bed rest (in days)	30 (22; 37)	21	30–45	90	21	21–28	35
Permissible degrees of hip flexion for meals (in degree)	30 (8; 41)	0	30	45	0	45	30
Antibiotic drugs used in immediate post-operative care	NA	FOX	AMC	TZP and anti MRSA	AMC or TZP	AMC or TZP	AMC
Mean duration of antibiotic treatment (in days)	44 (42; 49)	21–60	5	45	21–80	42	90–180
Success of treatment at 1 year (in %)	79 (9)	70	70	85	80	NA	90

Data as mean (SD) or as median (IQR).

*AMC* amoxicillin-clavulanate, *FOX* cefoxitine, *anti MRSA* anti Methicillin-resistant *Staphylococcus aureus* (either Daptomycin or Linezolid), *TZP* piperacillin-tazobactam, *MD* missing data, *NA* not applicable.

For sacral and ischial pressure injuries, the main surgical techniques included perforator flap (two centers), musculocutaneous flap (two centers), and a combination of both techniques (two centers). Trochanter pressure injuries were primarily treated with fascia lata flap (three centers), perforator flap (two centers), or a combination of both techniques (one center). Perineal pressure injuries were addressed using gracilis flap (two centers), or a combination of gracilis flap, perforator flap, and scrotal flap (four centers). Flap procedures were not performed during septic complications and were avoided during the acute phase of the bedsore.

Postoperative bedridden periods ranged from 21–45 days, consistently employing dynamic air pressure mattresses, with most centers implementing alternating postures, including prone position (three centers). Mealtime hip flexion (limited to 30 or 45°) was allowed in four centers but prohibited in two centers. Surgical stays ranged from returning to physical medicine and rehabilitation (PMR) service on day 0 to a maximum of 12 days.

The length of stay varied, with three centers opting for systematic hospitalization in a PMR service for the entire bedrest period (lasting 2–4 months), while a minority of people were allowed to return home with hospitalization at home. Seat recovery was conducted progressively across all centers, with air cushion usage being prevalent (5 out of 6 centers).

Preoperative recommendations included strict bedrest in four centers, whereas partial and fractional sitting were allowed in two centers under specific conditions, considering the person’s general condition and suitable cushion.

Regarding postoperative antibiotic treatment, there was variability among centers, with some using broad-spectrum antibiotics such as anti-pseudomonal beta-lactams (piperacillin-tazobactam) and anti Methicillin-resistant Staphylococcus aureus (MRSA) coverage (either linezolid, daptomycin, or vancomycin), while others employed amoxicillin-clavulanate only. The duration of antibiotic therapy ranged from 5–180 days. Suction drainage after surgery was consistently applied, but not all centers performed microbiological cultures. Most centers did not cultivate the drainage, while two centers conducted cultivation and adjusted antibiotic therapy based on microbiological results. Histology analysis was performed in most centers.

The overall self-reported cure rate at one year ranged from 70–90%.

## Discussion

Our national survey highlights the diverse practices across France regarding the medical and surgical management of osteomyelitis associated with pressure injuries in specialized centers, revealing significant variations among facilities.

The diagnostic criteria for osteomyelitis related to pressure injuries remain a major challenge, as it represents one of the primary risk factors for recurrence [7, 10]. Different diagnostic approaches are employed depending on the hospital and center as underline by previous surveys [6, 7]. In this study, the diagnosis of osteomyelitis was based on both clinical and microbiological criteria, in accordance with physician practice. Clinically, the diagnosis was supported by the presence of bone exposure on examination, while microbiological confirmation was obtained through the identification of microorganisms in intraoperative bone samples. Histopathological examination was performed in half of the centers but is not a routine practice in France.

Surgical techniques also vary widely between centers, primarily reflecting the preferences and practices of individual departments [6, 7, 10]. Musculocutaneous flaps offer effective cushioning of the affected area, potentially reducing or delaying recurrence risks. This approach is relatively quick and avoids the risk of venous congestion, but it results in a bulky scar. Conversely, perforator flaps, a newer technique, require a microsurgery-trained team and the use of an intraoperative Doppler device. While this method is more time-consuming and carries a risk of venous congestion, it offers other advantages. Importantly, previous surgical cohort studies have shown no clear association between the surgical technique used and recurrence rates [2, 7, 8]. Despite this surgical heterogeneity, none of the surveyed centers perform flap procedures during acute infections due to the high risk of failure in such situations [3].

Postoperative bedridden periods and permissible hip flexion angles vary significantly among centers. Currently, no data support the superiority of one strategy over another [2]. However, prolonged bedridden periods can lead to complications such as heart failure, thromboembolic events, and muscle deconditioning. Similarly, restricting hip flexion may complicate feeding, increasing the risk of aspiration pneumonia and malnutrition. These considerations are critical given the importance of nutritional status in optimizing recovery in this population. The variability in the length of hospital stays is primarily attributable to differences in local care pathways, which depend on the site’s network and resources. Interestingly, some centers have implemented early discharge protocols for select people with a favorable home environment. This strategy is particularly relevant given the current hospital bed shortages. The progressive reintroduction of seated positioning is a standard practice across all centers. This approach, combined with the use of appropriate cushions and seating systems, is crucial in promoting pressure injury healing.

The duration of antibiotic therapy remains a challenging issue, largely influenced by the quality of surgical debridement [4, 7, 11]. Nevertheless, minimizing unnecessary antibiotic use is essential in this vulnerable population, which already faces high antibiotic exposure and a significant risk of colonization and infection by MDROs [5]. In some cases, very short treatment durations have proven effective [3].

In this study, contrary to previous surveys, the main center implemented a very short course of antibiotics, suggesting that treatment practices could be standardized while significantly reducing antibiotic duration, given the comparable success rates across centers. Additionally, this study underscores the importance of both preoperative management and postoperative care, including strict bed rest, gradual mobilization, and coordinated discharge to designated care facilities. The originality of this work lies in its emphasis on the importance of a multidisciplinary approach, with rehabilitation physicians playing a central role in ensuring successful patient management.

## Conclusion

Our national survey highlights the diverse practices surrounding the medical and surgical management of osteomyelitis associated with pressure injuries in specialized centers, revealing considerable variability among facilities. Despite these heterogeneous management approaches, the overall cure rate remains high. However, given the vulnerability of this population, there is a pressing need for more robust data to develop standardized, evidence-based management protocols incorporating comprehensive evaluations.

## Supplementary information

Supplementary Information (Methods)

## Data Availability

The datasets generated during and/or analysed during the current study are available from the corresponding author on reasonable request.
