# Supplementary material for: Management of pressure injuries with associated osteomyelitis in people with spinal cord injury: a national survey of referral centers in France
Source: Spinal Cord. 2025 May 10;63(6):319–22. doi: 10.1038/s41393-025-01084-y (PMC12173937; doi:10.1038/s41393-025-01084-y)
Supplement: Supplementary file 1 — Supplementary Information (Methods) [file 41393_2025_1084_MOESM1_ESM.docx]

**SUPPLEMENTARY INFORMATION**

**METHODS: Questionnaire used for the National survey**

**Part I: General Questions / Surgery (40 questions, including 19 open-ended questions)**

- City of practice, team composition (multidisciplinary approach).
- Frequency of visits and staff meetings.
- Number of surgeries per year and one-year success rate.
- Patient characteristics: age, gender ratio, type of neurological condition.
- Type of surgical flap used for coverage depending on the location of pressure ulcers.
- Discharge modalities: Nursing Home, Rehabilitation and Aftercare Facility, Physical and Rehabilitation Medicine Unit, Specialized Care Home, Home Hospitalization, Private Registered Nurse.

**Part II: Rehabilitation Medicine (25 questions, including 9 open-ended questions)**

- Preoperative assessment performed: occupational therapy/urological assessment/nutritional assessment/excreta management.
- Seating assessment, evaluation of equipment (adapted or not).
- Postoperative instructions: strict bed rest or not, allowable hip flexion angle, and modalities for resuming sitting.

**Part III: Infectious Diseases (15 questions, including 8 open-ended questions)**

- Postoperative antibiotic protocol (empirical and targeted): drug, dosage, and duration.
- Use of Redon drains: indications, duration, and interpretation.
- Microbiological sampling: type and number of samples.
- Anatomopathological examination.
